# Effect of Relative Age on Gross Motor Coordination Development, Considering Biological Maturity and Sex

**DOI:** 10.3390/children12050619

**Published:** 2025-05-10

**Authors:** Xiaoyu Zhang, Gaël Ennequin, Anthony J. Blazevich, Sébastien Ratel

**Affiliations:** 1 AME2P, UFR STAPS, Université Clermont Auvergne, F-63000 Clermont-Ferrand, France; xiaoyu.zhang2@doctorant.uca.fr (X.Z.); gael.ennequin@uca.fr (G.E.); 2 Centre for Human Performance, School of Medical and Health Sciences, Edith Cowan University, Joondalup, WA 6027, Australia; a.blazevich@ecu.edu.au

**Keywords:** relative age effect, maturation, motor skill development, talent identification, youth athlete development

## Abstract

Objective: This study aimed to investigate the effect of relative age on gross motor coordination (GMC) development, taking into account biological maturity and sex. Methods: 729 participants aged 10 to 16 years completed three GMC tests newly designed to minimize the potentially confounding effects of physical fitness. The tests included a Hand-Foot coordination test, Dribbling-Targeting test (DT test), and a T balance and agility test (T test). Relative age was determined using birth quartiles: BQ_1_ (January–March); BQ_2_ (April–June); BQ_3_ (July–September); and BQ_4_ (October–December). Regarding biological maturity, the participants were categorized according to their estimated peak height velocity (pre- and post-PHV). Results: Relative age did not significantly impact the overall GMC score. In contrast, biological maturity emerged as a significant determinant of GMC, with post-PHV participants outperforming their pre-PHV counterparts in the three tests (*p* < 0.001). The results also showed a significant main effect of sex in the DT and T tests, i.e., in tasks involving dynamic coordination and agility, with boys consistently outperforming girls (*p* < 0.001). Significant biological maturity × sex interactions in the DT and T tests also indicated an advantage of boys over girls with advancing biological maturity (*p* < 0.05). Conclusions: these findings highlight the importance of considering biological maturity and sex rather than relative age in GMC development from childhood through adolescence.

## 1. Introduction

Gross motor coordination (GMC) represents the capacity to execute precise, smooth, and controlled body movements through the integrated function of the nervous and musculoskeletal systems. This fundamental ability significantly impacts children and adolescents’ athletic performance, daily functioning, and long-term physical health [1]. GMC constitutes a critical component of gross motor skill competency [2], encompassing both fundamental movement skills (e.g., locomotor skills as running, jumping, hopping, and galloping) and GMC but excluding physical capacities [3]. Research demonstrates that superior GMC not only provides children with competitive advantages in sports and games but also correlates with enhanced physical and mental health, higher levels of physical activity, and accelerated motor skill development [4,5]. Despite its importance, GMC development varies considerably among children, influenced by multiple factors, including biological maturity, sex, and relative age. While the relative age effect (RAE) has been extensively studied in sports [6,7], its role in GMC development warrants further exploration.

The RAE describes the advantage that chronologically older children within an age cohort possess over their younger peers in terms of physical fitness, motor skills, and skill acquisition [8]. This phenomenon emerges because children born earlier in the selection year typically exhibit advanced chronological age, more developed physical characteristics, and extended periods for growth and training than later-born peers [8,9]. In competitive sports environments, the RAE has been consistently documented, particularly among youth elite athletes, where relatively older individuals demonstrate higher rates of selection and advancement [10]. For example, talent identification processes in youth soccer, basketball, and ice hockey reveal significantly higher proportions of players born in the first and second birth quartiles (BQ_1_ and BQ_2_) than those born in the third and fourth quartiles (BQ_3_ and BQ_4_) [11,12,13]. However, in non-competitive contexts, specifically regarding GMC development in adolescents, the magnitude of influence of RAE on motor development remains ambiguous. Recent studies have demonstrated that students around the age of 13 years exhibit significant RAE in both physical and motor variables [14]. Conversely, Wattie, Tietjens, Schorer, Ghanbari, Strauss, Seidel, and Baker [15] found no significant RAE in seven of eight motor performance measures when studying German children aged 9–10 years. Some studies show that the RAE is more evident in early childhood and tends to decline with age due to reduced maturity gaps. For example, Nakata et al. [16] found a pronounced RAE in physical fitness among elementary school children, which weakened by junior high school. In contrast, Vučković et al. [17] reported a persistent RAE in certain motor abilities during adolescence. These inconsistencies, likely influenced by factors such as sex, skill type, or participation level, highlight the context-dependent nature of RAE and motivated this study.

During children’s and adolescents’ motor development, the RAE may be modulated by multiple factors, with biological maturity and sex being key factors. Biological maturity reflects individual variations in growth and development progression, potentially influencing motor ability acquisition [18]. During adolescence, the period around the peak height velocity (PHV) triggers physiological transformations that could alter RAE impact. Evidence indicates that children with advanced biological maturity typically outperform less mature counterparts in strength, speed, and coordination [19]. Additionally, sex appears to play a pivotal role in GMC development. Research predominantly suggests that boys outperform girls in coordination tasks, particularly those requiring strength and speed [20,21], although some studies report no sex differences [22] or even enhanced balance capabilities among girls [23,24]. While sex differences in GMC have been thoroughly studied, their interaction with RAE remains insufficiently characterized. Most RAE investigations have focused on male populations, showing stronger effects in boys than girls [25,26], while other studies revealed no discernable RAE in female athletes [27]. Specifically, although the RAE has been widely studied in the context of sports performance and participation, relatively few studies have explored its influence on the fundamental development of GMC across different stages of growth. Our study aims to fill this gap by examining not only the presence of RAE across a broad age range (from childhood into adolescence), but also by comparing its effects with those of biological maturity and sex—two key developmental factors known to influence GMC.

Therefore, the aim of this study was to investigate the RAE on GMC, taking into account biological maturity and sex. We hypothesized that between 10 and 16 years: (i) the RAE would be more pronounced in pre-pubertal children (pre-PHV) but would attenuate or disappear in post-pubertal children (post-PHV), (ii) boys would generally exhibit superior GMC to girls, with RAE exhibiting stronger effects in boys than girls, and (iii) biological maturity would significantly influence GMC, with more biologically mature individuals displaying superior motor performance.

## 2. Materials and Methods

### 2.1. Participants

**Figure 1:**
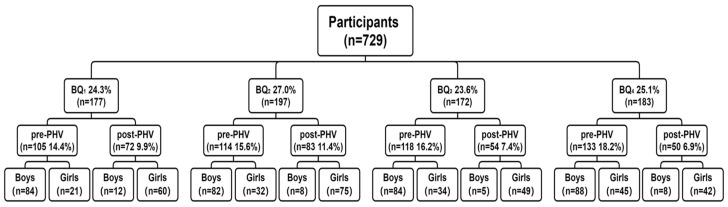
Flowchart of participant distribution by birth quarter (BQ_1_–BQ_4_), biological maturity (pre/post-PHV), and sex.

The lack of relevant data on the assessment of GMC according to RAE, biological maturity, and sex did not allow an *a priori* power analysis to be conducted to determine a target sample size. Therefore, we recruited as many participants as possible. A total of 729 participants (boys: *n* = 371; girls: *n* = 358) aged 10 to 16 years (Table 1) were recruited from elementary and middle schools in France. They were categorized according to their relative age, biological maturity, and sex (see Figure 1). Relative age was determined using the birth quartile method, which categorizes individuals into four groups based on their birth month: BQ_1_ (January–March), BQ_2_ (April–June), BQ_3_ (July–September), and BQ_4_ (October–December). Regarding biological maturity, the participants were categorized according to their estimated peak height velocity (see below for further details). All participants completed two physical education lessons per week (3–4 h) and two daily active play periods of 30 min each. The study was approved by the Lyon academy’s institutional review board (IA IPR 03052021) and all school principals. The study respected the ethical guidelines of the sixth Declaration of Helsinki. Written assent was obtained from all participants, and informed consent was provided by the parents or guardians of participants before testing.

### 2.2. Experimental Design

Participants completed testing across two sessions. Anthropometric measurements were completed and test procedures explained in the first session while GMC was assessed using three newly developed tests designed to minimize the influence of physical fitness in the second session (see below for details). All tests were conducted in school gyms during regular school hours between September 2021 and December 2024. Data collection was performed by trained physical education teachers from each participating school. To ensure that the results reflected innate motor coordination rather than performance acquired through repetition, no familiarization trials were conducted. Instead, all the procedures were demonstrated through standardized instructional videos, with a particular emphasis on common errors, allowing participants to mentally prepare and understand each task without prior physical practice. This approach was intended to minimize motor learning effects and better assess participants’ ability to execute novel motor tasks—thereby capturing aspects of motor intelligence. In the second session, participants completed the three GMC tests in a self-selected order, with two attempts per test and a mandatory two-minute rest between trials. Each session lasted approximately 20 min per student. All the data were independently verified by a research assistant who was not involved in the data collection process.

### 2.3. Anthropometry

Body mass (BM) was measured using a digital scale (TANITA, BC-545N, Tokyo, Japan) and standing height was assessed with a portable stadiometer (TANITA, HR001, Tokyo, Japan) with participants barefoot. Sitting height was measured using the same stadiometer, with participants seated on the floor with their back against a wall. All measurements were taken with participants wearing light clothing and without shoes.

### 2.4. Biological Maturity Assessment

Biological maturity was assessed from maturity offset (MO, i.e., years to (from) age at peak height velocity (APHV)) using chronological age, standing height, sitting height, and body mass. Its calculation was based on sex-specific regression equations according to the method proposed by Mirwald, Baxter-Jones, Bailey, and Beunen [28]. Participants were considered as pre-PHV (MO < 0) or post-PHV (MO > 0).

### 2.5. Gross Motor Coordination Assessment

#### 2.5.1. Hand-Foot Test (HF Test)

The HF test was designed to evaluate stationary coordination and balance. Participants stood on a stable 1 × 1-m square floor surface and were instructed to complete as many consecutive cycles as possible of four crossed touches between the hands and feet (two in front and two behind; see Supplementary File—Test S1) within 20 s. The test ended at the first error, such as the participant touching the square’s boundary lines, performing the wrong sequence, or failing to touch the foot with the hand. Performance was assessed by the number of correctly completed cycles and the time elapsed until the first mistake (maximum 20 s). One experimenter managed the timing while another recorded the number of successful cycles. A countdown (‘5-4-3-2-1-GO’) was given before each trial, with the stopwatch starting on “GO”. No verbal encouragement was provided to avoid interference. The score (S_HF_) was calculated by multiplying the number of correct cycles by the time. Each participant completed two trials with a 2-min rest between, and the best score was retained. Higher scores reflected better stationary balance, better coordination between upper and lower limbs, and faster execution.

#### 2.5.2. Dribbling-Targeting Test (DT Test)

The DT test was designed to assess coordination, balance, and ball control. Participants stood on a stable 1 × 1-m square floor surface and were instructed to complete as many consecutive test cycles as possible within 20 s. Each cycle involved four alternating right- and left-hand dribbles with a basketball, followed by a two-handed throw to a 0.5 × 0.5 m square target positioned 2.5 m away on a wall, and catching the ball with both hands directly or after a rebound (see Supplementary File—Test S2). The test ended at the first error, such as the participant touching the square’s boundary lines, failing to follow the dribble sequence, interrupting the dribble by catching the ball, touching the ball with a body part other than the hands, or missing the target. Performance indicators included the number of correctly completed cycles and the time elapsed until the first error (maximum 20 s). Test management, including time recording, cycle counting, pre-test countdown, and verbal encouragement, was identical to the HF test. The score (S_DT_) was calculated by multiplying the time by the number of successful cycles. Higher scores reflected better coordination, balance, and ball control at a high execution speed.

#### 2.5.3. T Test

The T test was designed to assess agility and balance in dynamic conditions. Participants aimed to achieve the highest number of foot-to-cone contacts within a 1 × 2-m “T”-shaped corridor in 20 s. Starting at point A facing two cones (see Supplementary File—Test S3), participants ran backwards to point B at the start signal, side-stepped to point C or D, and tapped the top of each cone with the outermost foot, side-stepped to the opposite cone and repeated the taps, returned to point B, and then sprinted forward to point A, tapping both cones with each foot. The test (and timing) was stopped at the first error, including when participants touched the lines marking the “T”-shaped zone, a cone was not touched, a cone was overturned, a cone was touched with the foot farthest from the cone (i.e., with the wrong foot), or a foot touched the ground between the two cones. Performance indicators were the number of correctly touched cones and the time until the first error (maximum 20 s). Test management, including time recording, cycle counting, pre-test countdown, and verbal encouragement, was identical to the HF test. The score (S_T_) was calculated by multiplying the time by the number of correct touches. Higher scores indicated better agility, balance, and speed in dynamic conditions.

#### 2.5.4. Total Gross Motor Coordination Score (S_GMC_)

A score (S) of gross motor coordination (S_GMC_) was subsequently calculated by adding the score of each test (S_HF_ + S_DT_ + S_T_).

### 2.6. Statistical Analysis

The normality of data distribution and homogeneity of variances were assessed using Shapiro–Wilk and Bartlett’s tests, respectively. A three-way ANOVA was performed to examine the effects of relative age, biological maturity, and sex as well as their interactions on GMC scores (S_GMC_, S_HF_, S_DT_, S_T_). When significant main or interaction effects were detected, Fisher’s LSD post-hoc tests were used to make pairwise comparisons. Effect sizes were reported as partial eta-squared (η_p_^2^) and classified as small (∼0.01), moderate (∼0.06), or large (≥0.14) [29]. Statistical significance was set at *p* < 0.05. All analyses were conducted using SPSS 20.0 (IBM Corp., Somers, NY, USA), and the results are presented as mean ± SD in the text and tables.

## 3. Results

Table 1 displays the physical characteristics and GMC performance scores for each group. There were no significant relative age × biological maturity × sex interaction effects for chronological age, standing height, or body mass.

**Figure 2:**
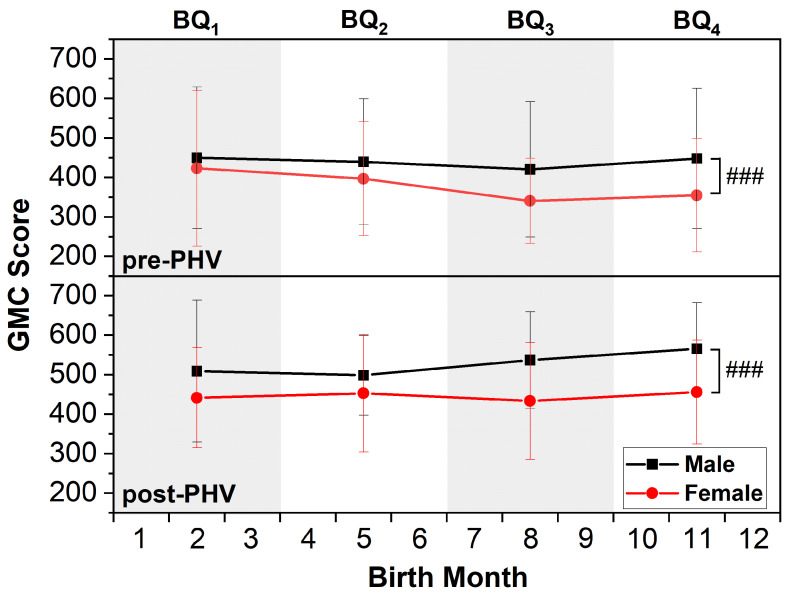
Mean (SD) GMC scores according to biological maturity, relative age, and sex. GMC score represents the sum of the scores from the three tests. Relative age was categorized into BQ_1_ (January–March), BQ_2_ (April–June), BQ_3_ (July–September), and BQ_4_ (October–December) based on birth month. GMC score increases significantly from before (pre-PHV) to after (post-PHV) peak height velocity periods (*p* < 0.001). ### indicates a significant difference between sexes (male vs. female) at *p* < 0.001.

ANOVA revealed no significant main effect of relative age (*F*_(3,729)_ = 0.430, *p* = 0.732, η_p_^2^ = 0.002) or interaction effects between relative age, biological maturity, or sex for the total GMC score (S_GMC_) (*F*_(3,729)_ = 0.190, *p* = 0.903, η_p_^2^ = 0.001). However, there were significant main effects of biological maturity (*F*_(1,729)_ = 23.446, *p* < 0.001, η_p_^2^ = 0.032) and sex (*F*_(1,729)_ = 16.920, *p* < 0.001, η_p_^2^ = 0.023) on S_GMC_. Specifically, participants classified as post-PHV outperformed pre-PHV (*p* < 0.001). Boys also demonstrated significantly higher GMC scores than girls (*p* < 0.001) (Figure 2).

For the individual GMC tests, no significant main or interaction effects were observed for S_HF_, indicating that performance was unaffected by relative age, biological maturity, or sex. Similarly, no effect of relative age was found in any of the individual tests (S_HF_: *F*_(3,729)_ = 0.330, *p* = 0.804, η_p_^2^ = 0.001; S_DT_: *F*_(3,729)_ = 0.554, *p* = 0.646, η_p_^2^ = 0.002; S_T_: *F*_(3,729)_ = 0.471, *p* = 0.702, η_p_^2^ = 0.002). In contrast, S_DT_ and S_T_ showed significant main effects of biological maturity (S_DT_: *F*_(1,729)_ = 8.454, *p* = 0.004, η_p_^2^ = 0.012; S_T_: *F*_(1,729)_ = 31.195, *p* < 0.001, η_p_^2^ = 0.042) and sex (S_DT_: *F*_(1,729)_ = 7.686, *p* = 0.006, η_p_^2^ = 0.011; S_T_: *F*_(1,729)_ = 16.593, *p* < 0.001, η_p_^2^ = 0.023). Boys performed significantly better than girls, and post-PHV participants outperformed pre-PHV participants. Additionally, significant biological maturity × sex interaction effects were found for S_DT_ (*F*_(1,729)_ = 6.581, *p* = 0.011, η_p_^2^ = 0.009) and S_T_ (*F*_(1,729)_ = 5.566, *p* = 0.019, η_p_^2^ = 0.008), revealing an advantage of boys over girls with advancing biological maturity (*p* < 0.05).

## 4. Discussion

This study investigated the effects of relative age, biological maturity, and sex on gross motor coordination (GMC) from childhood through adolescence. The findings did not support the hypothesis that relative age would significantly impact GMC performance, as no main or interaction effects of relative age were observed. Instead, the results demonstrated that biological maturity (post-PHV > pre-PHV) and sex (boys > girls) served as primary determinants of GMC scores. Furthermore, no interaction effects between relative age, biological maturity, and sex were detected, suggesting that the developmental trajectories of GMC remained unaffected by birth quartile when accounting for biological maturity and sex. These findings suggest that GMC development is strongly associated with biological maturity and sex, while relative age exerts a minimal influence on motor competency from late childhood through adolescence.

### 4.1. Relative Age and GMC

The results revealed no significant effect of relative age on GMC, with neither main nor interaction effects being observed for relative age across overall GMC scores or individual subtest performances. This finding is consistent with previous research suggesting that the relative age influence may progressively diminish with advancing chronological age [14,16]. However, some investigations have reported contrasting evidence, demonstrating significant differences in motor competence associated with relative age, though these findings predominantly emerge from populations younger than 10 years old [30,31].

Generally, the relative age effect (RAE) manifests more prominently in sports where physical advantages, including speed, strength, or endurance, play a crucial role [11]. Yet the GMC assessments employed in this study were specifically designed to minimize the influence of physical fitness while emphasizing neuromuscular coordination development. The absence of significant relative age effects may therefore relate to the fundamental characteristics of GMC, which likely depend more heavily on long-term sports technology, experience, nervous system maturity, and individual perception–action integration ability rather than physical attributes alone. Moreover, biological maturity may mask relative age influences on GMC. Previous research has highlighted the critical role of biological maturity in adolescent athletic performance [32]. Sherar, Baxter-Jones, Faulkner, and Russell [19] demonstrated that post-pubertal biological maturity exerted a greater influence than relative age effects on athletic performance. During adolescence, individual maturation differences may reduce relative age effects, making them less pronounced. Consequently, biological maturity may constitute a more critical factor than birth month in determining an individual’s motor development from late childhood or early adolescence onward.

### 4.2. Biological Maturity and Sex in GMC

Unlike the relative age effect, this study further supports the significant influences of biological maturity and sex on GMC. Biological maturity plays a crucial role in motor coordination development through its close association with neuromuscular efficiency, proprioceptive function, and motor control refinement [32]. The findings of this study indicate that post-PHV participants outperformed their pre-PHV counterparts across all GMC tests, suggesting that advanced biological maturity confers advantages in motor competency. This is consistent with the previous investigations [19] and may reflect that more mature individuals typically exhibit greater muscle strength, faster reaction times, and more precise motor control [33]. Furthermore, research suggests that children who reach their PHV earlier tend to outperform their less mature peers in coordination tasks. This advantage may stem from improvements in motor control and enhanced muscle strength resulting from rapid physical growth throughout adolescence [34].

Neuromuscular adaptation may also play a role. Children with advanced biological maturity typically possess a more developed central nervous system (CNS), facilitating more efficient neural pathways. They also tend to exhibit superior sensorimotor integration, further supporting motor coordination [35]. These factors may explain why adolescents with higher biological maturity demonstrated superior performance in GMC tests in this study.

These findings further indicated that boys demonstrated significantly higher GMC scores than girls, which is consistent with previous research [21,24,36]. This disparity may be attributed to the combined influence of physiological and sociocultural factors. From a physiological perspective, boys generally possess greater muscle mass, elevated androgen hormone levels (e.g., testosterone), and superior neuromuscular control, all of which contribute to improved athletic performance and physical ability [37]. In contrast, some studies have reported opposite findings, suggesting that girls may possess advantages in flexibility and balance [38]. However, these attributes may not necessarily translate into higher GMC scores, particularly in tasks involving dynamic motor activities.

Furthermore, the gender differences in GMC may be influenced by environmental and cultural factors. Boys are frequently encouraged to participate in more intense and competitive physical activities, such as football (all codes) and basketball, which foster the development of motor skill and coordination development [39]. Conversely, girls may encounter fewer opportunities to engage in structured physical activities, limiting their motor learning experiences [40]. This difference in sports participation may partially explain the male superiority in GMC observed in this study.

### 4.3. Subtest Analysis

Subtest analyses revealed that the Hand-Foot test (HF test) showed no significant main or interaction effects for relative age, biological maturity, or sex. This result may reflect that relative static balance and coordination tasks tend to stabilize earlier in development and are less influenced by biological maturity and sex than whole-body dynamic coordination tasks [41]. In contrast, the ball Dribbling-Targeting test (DT test) and T balance and agility test (T test) demonstrated significant main effects for biological maturity and sex, along with significant interactions between these factors. These findings suggest that more dynamic motor tasks, even in our modified test forms, remain sensitive to developmental variation. Notably, the interaction effects imply that boys benefit more from biological maturation than girls, leading to an increasing performance gap during adolescence [34].

This interaction has important implications for school-based physical education and program planning. As boys and girls do not follow identical developmental trajectories, applying uniform standards or task designs may unintentionally disadvantage less mature students, particularly girls, during key developmental windows. Recognizing the maturity × sex interaction allows educators and practitioners to design more inclusive and developmentally appropriate physical activities. For example, sex-specific benchmarks, differentiated difficulty levels, or maturity-informed groupings can help ensure fair evaluation and equal opportunities for motor skill acquisition, participation, and advancement in school sports and physical education.

### 4.4. Individual Differences in GMC

There was considerable within-group variability in GMC, reflecting significant inter-individual differences (see SD in Figure 2). This indicates that some children may inherently demonstrate superior motor skill proficiency, which could prove critical for talent identification. Early identification of children with advanced or delayed GMC may inform the design of individualized training programs to enhance their abilities and support long-term athletic development [42], and future research is warranted to explore this possibility.

### 4.5. Strengths and Limitations

In this study, factors such as sports experience and training background, which could also contribute to the reduction of the RAE, were not considered. Additionally, environmental and cultural factors such as socio-economic status (SES), which may influence access to physical activity opportunities and consequently motor development, were not formally assessed or controlled for. Future investigations that include these elements may provide a more complete picture of the various influences on GMC. Another point to consider is the use of newly developed tests without formal validation procedures. While such validation is generally recommended, our primary objective was to minimize potential learning effects in order to assess innate GMC and motor intelligence rather than performance acquired through repetition. Additionally, we developed novel tests designed to reduce the confounding influence of physical fitness components, such as speed, strength, and endurance, which are embedded in widely used tools like the Körperkoordinationstest Für Kinder (KTK) [18]. Notably, two of the four KTK sub-tests—the sideways jumping test and the hopping test—rely heavily on speed and power, making them less suitable for isolating GMC [18]. Validating our tests against such instruments would have reintroduced the very confounding factors we aimed to eliminate. Nevertheless, our tests demonstrated sensitivity to key developmental factors, including age, sex, and biological maturity. Moreover, the observed patterns aligned with findings from the existing literature, further supporting their construct validity.

One major strength is the implementation of newly developed tests designed to minimize the confounding influence of physical fitness, focusing more specifically on GMC. Furthermore, this is the first study to simultaneously consider sex, biological maturity, and relative age, offering a more comprehensive insight into factors influencing GMC. Lastly, the sample spanned a wide age range from childhood through adolescence, facilitating a deeper understanding of GMC development and providing valuable reference data for future research.

## 5. Conclusions

This study investigated the effects of relative age, biological maturity, and sex on GMC. The results indicated that relative age did not significantly influence GMC, while boys outperformed girls in all tests and biological maturity emerged as a key determinant of GMC development from childhood though adolescence. It should be noted that a significant effect of both biological maturity and sex were observed in the ball Dribbling-Targeting test (DT test) and T balance and agility test (T test), which both involve dynamic coordination and agility. This finding suggests that as children mature biologically, their performance in tasks requiring coordination and agility improves, with boys generally outperforming girls. Practically, this highlights the importance of considering biological maturity when assessing and training motor skills in youth sports. Coaches and trainers should account for these differences to ensure fair talent identification and appropriate training interventions. In contrast, relative age impact appears to gradually diminish with increasing age, especially around the pubertal transition when individual differences in biological maturity become more critical in determining motor performance. These findings underscore the importance of considering biological maturity rather than relative age in talent identification and youth athlete development programs from late childhood through adolescence.

## 6. Practical Applications

These findings offer valuable insights for youth talent identification and sports training programs, with important implications for practice.

First, the results highlight the significant role of biological maturity in GMC development, suggesting that selection processes should prioritize biological maturity over relative age assessments from late childhood through adolescence. This approach could mitigate the risk of overlooking potential talents due to relative age bias, thereby promoting more equitable talent identification.

Second, the results emphasize the importance of individualized training approaches in youth sports. Programs tailored to biological maturity may optimize GMC development and reduce potential disadvantages among late-maturing children. Moreover, the observed sex differences suggest that girls may require more opportunities for physical activity and targeted training to bridge performance gaps in dynamic coordination and agility tasks. Sports education and training institutions should implement inclusive policies that encourage female participation in diverse physical activities to enhance motor skill development. Future sports development initiatives should adopt a multifaceted approach that integrates biological, psychological, and social factors to foster long-term athletic development and ensure fairness in talent selection.

## Figures and Tables

**Table 1:** Participants’ physical characteristics and gross motor coordination (GMC) scores.

		BQ_1_ (*n* = 177) ^1^		BQ_2_ (*n* = 197) ^2^		BQ_3_ (*n* = 172) ^3^		BQ_4_ (*n* = 183) ^4^	
		pre-PHV (*n* = 105)	post-PHV (*n* = 72)	pre-PHV (*n* = 114)	post-PHV (*n* = 83)	pre-PHV (*n* = 118)	post-PHV (*n* = 54)	pre-PHV (*n* = 133)	post-PHV (*n* = 50)
Age (years)	Girls	12.0 ± 0.4	13.0 ± 1.0	12.0 ± 0.3	12.8 ± 1.0	11.7 ± 0.3	12.7 ± 1.1	11.6 ± 0.1	12.9 ± 1.1
	Boys	12.5 ± 0.7	14.8 ± 0.5	12.2 ± 0.7	14.7 ± 0.5	12.1 ± 0.8	14.6 ± 0.5	11.9 ± 0.8	13.9 ± 0.5
Height (cm)	Girls	149.3 ± 3.6	160.0 ± 5.9	147.3 ± 5.0	161.0 ± 6.6	149.0 ± 4.8	158.2 ± 5.5	150.0 ± 5.8	160.3 ± 5.4
	Boys	153.8 ± 8.9	173.5 ± 8.6	153.7 ± 8.6	175.4 ± 6.4	152.5 ± 8.0	172.2 ± 8.1	152.2 ± 7.9	173 ± 3.0
Body mass (kg)	Girls	37.0 ± 5.2	50.3 ± 11.7	36.0 ± 4.6	50.6 ± 9.8	36.8 ± 5.3	48.8 ± 6.2	39.6 ± 5.8	52.6 ± 11.1
	Boys	44.7 ± 12.0	56.9 ± 8.5	43.5 ± 10.2	57.8 ± 6.1	42.7 ± 10.4	59.8 ± 7.7	42.4 ± 8.0	62.8 ± 8.9
MO (years) ^5^	Girls	−0.35 ± 0.25	0.95 ± 0.69	−0.56 ± 0.34	0.89 ± 0.72	−0.57 ± 0.28	0.72 ± 0.77	−0.51 ± 0.33	0.95 ± 0.67
	Boys	−1.64 ± 0.77	0.93 ± 0.74	−1.72 ± 0.71	1.06 ± 0.69	−1.92 ± 0.80	0.54 ± 0.65	−1.99 ± 0.73	0.66 ± 0.31
APHV (years) ^6^	Girls	12.0 ± 0.5	12.0 ± 0.6	12.3 ± 0.6	11.9 ± 0.6	12.1 ± 0.5	11.9 ± 0.6	11.8 ± 0.6	12.0 ± 0.6
	Boys	14.1 ± 0.7	13.9 ± 0.6	13.9 ± 0.7	13.6 ± 0.7	13.9 ± 0.8	14.1 ± 0.7	13.8 ± 0.7	13.2 ± 0.3
HF Test Score ^7^	Girls	164 ± 92	146 ± 67	134 ± 82	146 ± 75	113 ± 78	150 ± 78	137 ± 83	141 ± 71
	Boys	156 ± 88	154 ± 99	157 ± 86	131 ± 67	146 ± 81	196 ± 84	147 ± 94	173 ± 77
DT Test Score ^8^	Girls	85 ± 49	118 ± 50	85 ± 44	111 ± 48	76 ± 47	110 ± 49	78 ± 49	111 ± 35
	Boys	113 ± 53	108 ± 46	114 ± 50	113 ± 32	110 ± 52	97 ± 26	110 ± 47	135 ± 21
T Test Score	Girls	173 ± 109	177 ± 69	178 ± 94	195 ± 87	152 ± 89	173 ± 84	140 ± 91	204 ± 74
	Boys	180 ± 103	247 ± 103	168 ± 95	255 ± 49	165 ± 107	244 ± 30	191 ± 98	258 ± 116
GMC Score ^9^	Girls	423 ± 197	441 ± 127	397 ± 144	453 ± 148	341 ± 107	433 ± 148	355 ± 144	456 ± 132
	Boys	450 ± 179	509 ± 179	439 ± 160	498 ± 100	420 ± 171	536 ± 122	448 ± 177	566 ± 116

Data are presented as mean ± standard deviation. Abbreviations: ^1^ first birth quartile; ^2^ second birth quartile; ^3^ third birth quartile; ^4^ fourth birth quartile; ^5^ maturity offset; ^6^ age at peak height velocity; ^7^ Hand-Foot test score; ^8^ Dribbling-Targeting test score; ^9^ Gross Motor Coordination score (sum of the scores of the 3 tests).

## Data Availability

The data that support the findings of this study are available from the corresponding author upon reasonable request.

## Supplementary Materials

The following supporting information can be downloaded at: https://www.mdpi.com/article/10.3390/children12050619/s1, Test S1: Participants performed as many movement cycles as possible in 20 s on a stable surface within a 1 m × 1 m marked square, adhering to the ‘contact rule’. Each cycle consisted of four alternating foot and hand touches. The test was conducted twice consecutively, and the best score was recorded. The timing started with the participant’s first movement. Test S2: Participants completed as many ball-handling cycles as possible in 20 s, including four alternating right/left-hand dribbles while staying within the designated zone, a throw at a vertical target (one or two hands allowed), and retrieving the ball with or without a bounce without leaving the zone. The test was performed twice consecutively, with the best score recorded. The timing began with the participant’s first movement. Test S3: Participants performed as many foot/cone touches as possible in 20 s within a marked area on a stable surface, following the ’contact rule’. Starting at point A with their shoulders facing the cones, participants moved backward to point B, used side-steps to touch the top of two cones at points C and D with the outer foot, returned to point B using side-steps, and then ran forward to point A to touch the top of both cones with either foot. The cones were spaced 50 cm apart, positioned 25 cm from the line, and measured 20–30 cm in height. The test was conducted twice consecutively, with the best score recorded. The timing began with the participant’s first movement.
