# Supplementary material for: Effect of Relative Age on Gross Motor Coordination Development, Considering Biological Maturity and Sex
Source: Children (Basel). 2025 May 10;12(5):619. doi: 10.3390/children12050619 (PMC12110117; doi:10.3390/children12050619)
Supplement: Supplementary file 1 [file children-12-00619-s001.zip › children-3610717-supplementary.pdf]

Supplementary Materials

Test S1

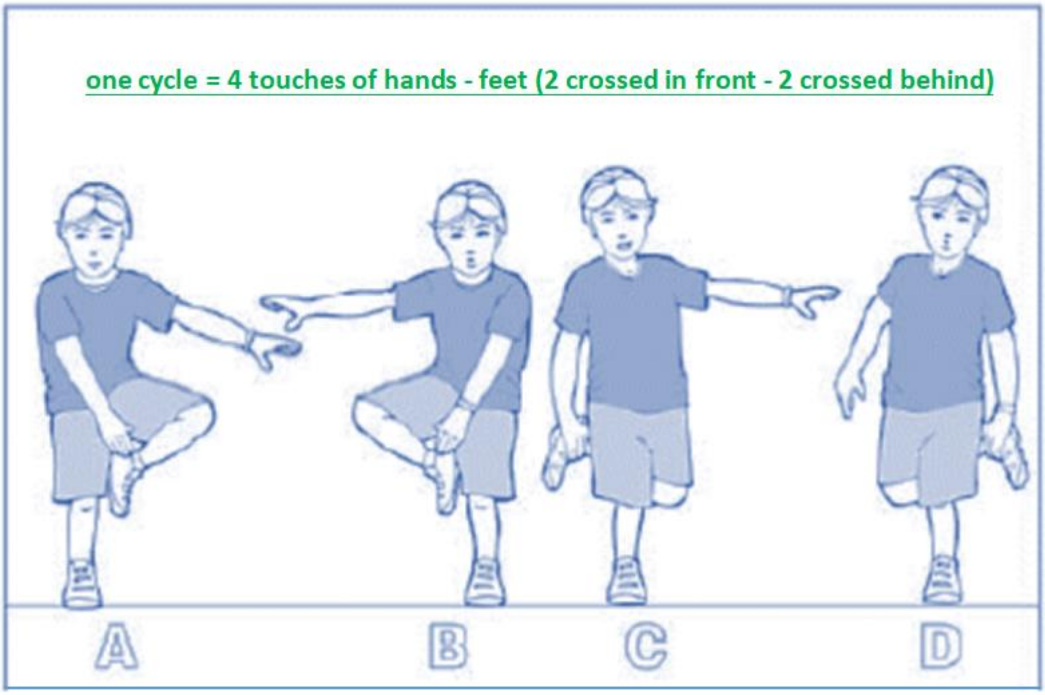

Participants performed as many movement cycles as possible in 20 seconds on a stable surface within a 1 m × 1 m marked square, adhering to the 'contact rule.' Each cycle consisted of four alternating foot and hand touches. The test was conducted twice consecutively, and the best score was recorded. Timing started with the participant's first movement.

Test S2

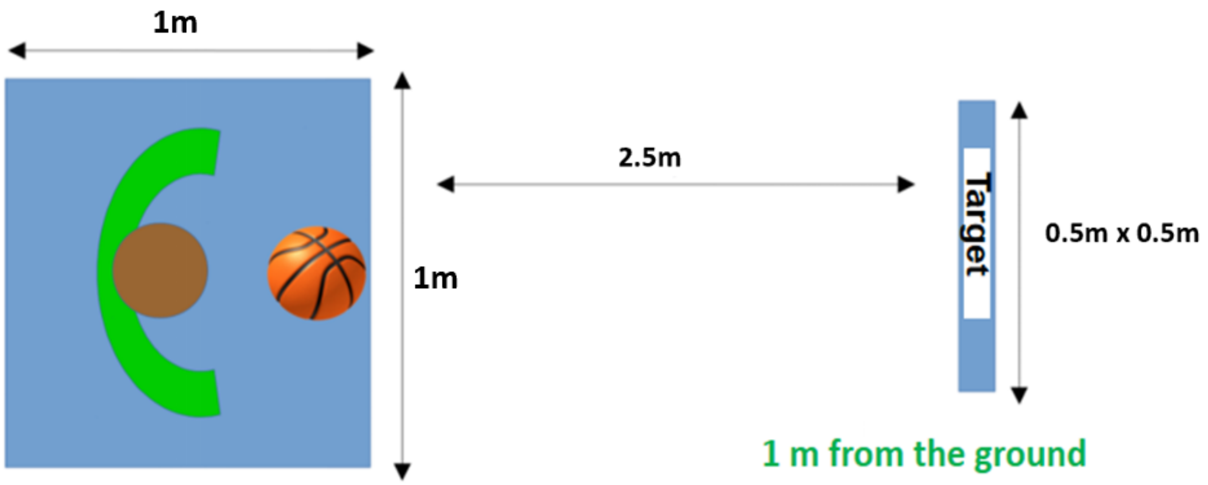

Participants completed as many ball-handling cycles as possible in 20 seconds, including four alternating right/left-hand dribbles while staying within the designated zone, a throw at a vertical target (one or two hands allowed), and retrieving the ball with or without a bounce without leaving the zone. The test was performed twice consecutively, with the best score recorded. Timing began with the participant's first movement.

Test S3

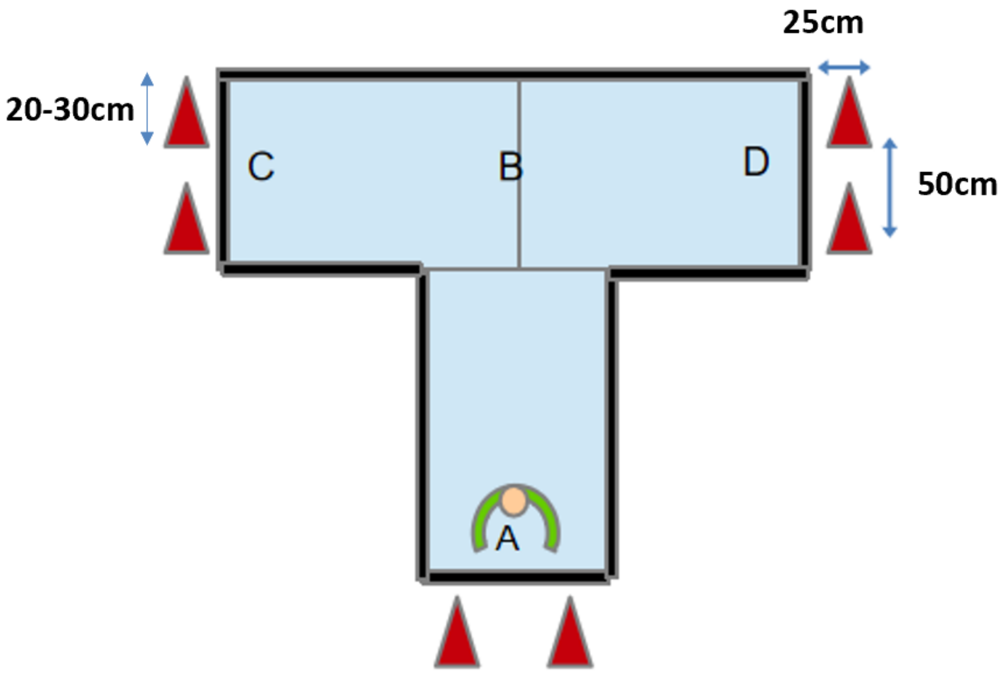

Participants performed as many foot/cone touches as possible in 20 seconds within a marked area on a stable surface, following the 'contact rule.' Starting at point A with shoulders facing the cones, participants moved backward to point B, used side-steps to touch the top of two cones at points C and D with the outer foot, returned to point B using side-steps, and then ran forward to point A to touch the top of both cones with either foot. Cones are spaced 50 cm apart, positioned 25 cm from the line, and measure 20-30 cm in height. The test was conducted twice consecutively, with the best score recorded. Timing began with the participant's first movement.
